# Effects of Long-Term Treatment with Ranirestat, a Potent Aldose Reductase Inhibitor, on Diabetic Cataract and Neuropathy in Spontaneously Diabetic Torii Rats

**DOI:** 10.1155/2013/175901

**Published:** 2013-03-13

**Authors:** Ayumi Ota, Akihiro Kakehashi, Fumihiko Toyoda, Nozomi Kinoshita, Machiko Shinmura, Hiroko Takano, Hiroto Obata, Takafumi Matsumoto, Junichi Tsuji, Yoh Dobashi, Wilfred Y. Fujimoto, Masanobu Kawakami, Yasunori Kanazawa

**Affiliations:** ^1^Department of Ophthalmology, Jichi Medical University, Saitama Medical Center, 1-847 Amanuma-cho, Omiya-ku, Saitama-shi 330-8503, Japan; ^2^Pharmacology Research Laboratories, Dainippon Sumitomo Pharma Co., Ltd., Osaka 554-0022, Japan; ^3^Department of Integrated Medicine I, Jichi Medical University, Saitama Medical Center, Saitama 330-8503, Japan; ^4^Division of Metabolism, Endocrinology and Nutrition, University of Washington School of Medicine, Seattle, WA, USA

## Abstract

We evaluated ranirestat, an aldose reductase inhibitor, in diabetic cataract and neuropathy (DN) in spontaneously diabetic Torii (SDT) rats compared with epalrestat, the positive control. Animals were divided into groups and treated once daily with oral ranirestat (0.1, 1.0, 10 mg/kg) or epalrestat (100 mg/kg) for 40 weeks, normal Sprague-Dawley rats, and untreated SDT rats. Lens opacification was scored from 0 (normal) to 3 (mature cataract). The combined scores (0–6) from both lenses represented the total for each animal. DN was assessed by measuring the motor nerve conduction velocity (MNCV) in the sciatic nerve. Sorbitol and fructose levels were measured in the lens and sciatic nerve 40 weeks after diabetes onset. Cataracts developed more in untreated rats than normal rats (*P* < 0.01). Ranirestat significantly (*P* < 0.01) inhibited rapid cataract development; epalrestat did not. Ranirestat significantly reversed the MNCV decrease (40.7 ± 0.6 m/s) in SDT rats dose-dependently (*P* < 0.01). Epalrestat also reversed the prevented MNCV decrease (*P* < 0.05). Sorbitol levels in the sciatic nerve increased significantly in SDT rats (2.05 ± 0.10 nmol/g), which ranirestat significantly suppressed dose-dependently, (*P* < 0.05, <0.01, and <0.01); epalrestat did not. Ranirestat prevents DN and cataract; epalrestat prevents DN only.

## 1. Introduction

Diabetes recently has reached almost epidemic levels worldwide. The major problem associated with diabetes is its complications, that is, diabetic retinopathy (DR) and cataract, diabetic neuropathy (DN), and nephropathy. DR is a leading cause of visual loss and blindness in adults [1]. Clinical trials have shown that intensive glycemic control reduces the incidence and progression of DR [2, 3]. Other metabolic factors, such as blood pressure [4] and hyperlipidemia [5, 6], also affect the development of DR. Multifactorial intensive treatment of these metabolic disorders and hyperglycemia prevent diabetic complications. However, many patients still develop serious complications, despite the recent availability of a variety of new antidiabetic drugs.

Very few drugs can directly prevent diabetic complications independent of the glucose levels. The metabolic changes accompanying hyperglycemia, such as increased activity of the polyol pathway [7], activation of protein kinase C (PKC) [8], increased oxidative stress [9], leukocyte adhesion to the endothelial cells [10], and accumulation of advanced glycation end products (AGEs) [11], are considered to be related to the development and progression of diabetic complications including ocular complications. In particular, the polyol pathway is correlated strongly with other complications including oxidative stress, activation of PKC, and accumulation of AGEs that lead to induction of vascular endothelial growth factor (VEGF). VEGF is the most important factor that induces retinal neovascularization. Accordingly, the polyol pathway is the most attractive target for adjunctive treatment to prevent diabetic ocular complications and DN. A key enzyme in the polyol pathway is aldose reductase (AR), which is found in the retina, lens, and Schwann cells of the peripheral nerves [12]; AR inhibitors (ARIs) have been reported to slow thickening of the basement membrane of the retinal capillaries and progression of diabetic cataract in experimental studies [13]. Based on favorable results in experimental studies using the ARIs [14], a clinical trial of an ARI, sorbinil, was conducted [15], but the drug did not have a relevant inhibitory effect on the development of DR, and enthusiasm for its clinical application for ocular complications waned. However, our previous study showed a strong preventative effect of an ARI, fidarestat, on the development of DR in spontaneously diabetic Torii (SDT) rats [16]. We recently confirmed that a newly developed ARI, ranirestat, is even more effective in preventing development of DR in SDT rats [17]. In the current study, we evaluated the effect of ranirestat on cataract and DN in SDT rats compared with the commercially available epalrestat. 

## 2. Methods

### 2.1. Animals

The care and handling of animals were in accordance with the Association for Research in Vision and Ophthalmology Statement for the Use of Animals in Ophthalmic and Visual Research and the Jichi Medical University Animal Care and Use Committee. Male SDT rats and Sprague-Dawley (SD) rats were obtained from CLEA, Inc. (Tokyo, Japan). All SDT rats were confirmed to be diabetic based on a nonfasting blood glucose concentration exceeding 350 mg/dL. All rats were fed standard rat chow (CRF-1, Oriental Yeast, Inc., Tokyo, Japan) with or without ARI. The ranirestat-treated rats were treated once daily with oral ranirestat; the epalrestat-treated rats were fed chow containing epalrestat at the onset of diabetes; and SD rats and untreated SDT rats were fed chow without an ARI. Epalrestat, an ARI commercially available in Japan, served as a positive control.

The animals were divided into six groups as follows: normal SD rats (*n* = 8), untreated SDT rats (*n* = 9), ranirestat-treated (0.1 mg/kg/day for 40 weeks) SDT rats (*n* = 7), ranirestat-treated (1.0 mg/kg/day for 40 weeks) SDT rats (*n* = 8), ranirestat-treated (10.0 mg/kg/day for 40 weeks) SDT rats (*n* = 7), and epalrestat-treated (100 mg/kg/day for 40 weeks) SDT rats (*n* = 8).

### 2.2. Measurement of Body Weight, Blood Glucose, and Glycated Hemoglobin

Body weight, blood glucose, and glycated hemoglobin (HbA1c) were measured once monthly. Blood samples were collected from the tail vein of nonfasting rats to measure plasma glucose and HbA1c. Blood glucose was measured with a glucose analyzer (Antosense, Bayer-Sankyo, Tokyo, Japan). HbA1c was measured using an automated glycohemoglobin analyzer (HLC-723GHb V, Tosoh Corporation, Tokyo, Japan).

### 2.3. Ocular Histopathology

Under deep anesthesia induced by an intraperitoneal injection of pentobarbital sodium (25 mg/kg body weight, Nembutal, Dainihonseiyaku, Osaka, Japan), the eyes were enucleated for conventional histopathologic studies and placed in a fixative (Superfix KY-500, Kurabo, Japan). The fixed eyes were washed in 0.1% mol/L cacodylate buffer and embedded in paraffin. The paraffin block was sectioned to 4 *μ*m and stained with hematoxylin and eosin for conventional histopathologic examination. The immunohistochemical procedures were based on the standard avidin-biotin horseradish peroxidase method using each antibody and developed with AEC Substrate Chromogen (DakoCytomation, Carpinteria, CA, USA). N*ε*-(Carboxymethyl)lysine (CML) was immunostained with a monoclonal antibody for human AGEs (1 : 50 dilution for CML, TransGenic Inc., Kumamoto, Japan). Bovine serum was used as a primary antibody for negative control of the immunostaining. 

### 2.4. Biomicroscopy of Cataract

The pupils were fully dilated with a topical ophthalmic solution containing tropicamide 5% and phenylephrine hydrochloride 5%, and the anterior segment including the lens was observed and photographed in both eyes of all rats. The degree of lens opacification was graded as follows: 0, clear normal lens; 1, slight opacity in the cortical layers; 2, diffuse opacity in the cortical layer; and 3, mature milky cataract. The opacity scores of both lenses of each animal were added and the sum was used as the total cataract score (0–6) for both eyes. Biomicroscopic slit-lamp examinations were performed at 15 and 40 weeks after the onset of the diabetes.

### 2.5. Measurement of Motor Nerve Conduction Velocity (MNCV)

The MNCV in the rat sciatic nerve was measured using the same method of previous report [18]. The rats were restrained in a prone position and anesthetized by continuous inhalation of 2.5% halothane gas. A constant rectal temperature of 37.5°C to 38.5°C was maintained by a temperature control system (ATB-1100, Nihon Kohden, Tokyo, Japan). The right sciatic nerve was used as the proximal stimulus point (S1), and the ankle region of the right tibial nerve was used as the distal stimulus point (S2). An active needle electrode (negative) was inserted at S1 and S2, and a reference electrode (positive) was inserted about 1 cm from S1 toward the spine. For recording, the active and reference electrodes were each inserted shallowly into the right plantar muscle. Using an induced potential detector (MEB-7202, Nihon Kohden), S1 and S2 were stimulated with single rectangular pulses (duration, 0.1 ms; current: supramaximal, 1.0–4.2 mA), and changes in the action potential were recorded. The proximal and distal latencies from stimulation of S1 and S2, respectively, to the rise of the action potentials (*t*_1_ and *t*_2_ in ms), respectively, and the distance between S1 and S2 (*d* in mm) were measured. The MNCV then was calculated using the following equation: MNCV  (m/s) = *d*/(*t*_1_ − *t*_2_).

### 2.6. Measurement of Sorbitol and Fructose

The rats were anesthetized with 50 mg/kg of intraperitoneal sodium pentobarbital (Abbott, Abbott Park, IL, USA), and the sciatic nerve, lens, and retina were removed, promptly cooled with liquid nitrogen, and stored at −50°C. Sorbitol and fructose contents in the sciatic nerve and lens tissues were determined by the method of Liang et al. [8]. Lens and sciatic nerves were homogenized with water at a concentration of 10 mg/mL, extracted with methanol and centrifuged (4°C, 10000 rpm, 1 min). One mL of the supernatant was applied to an InertSep SAX/SCX (50 mg/50 mg/1 mL) cartridge (GL Siences, Inc., Tokyo, Japan). The eluate was evaporated to dryness under a stream of nitrogen at 40°C. The residues were dissolved in 200 *μ*L of the mixture of acetonitrile/water (9 : 1, v/v). Then sorbitol and fructose contents were determined with the LC/MS/MS system which consisted of an SIL-HTC and LC-10A (Shimadzu Corp., Kyoto, Japan) and the API4000 tandem mass spectrometer (Applied Biosystems/MDX SCIEX, MA, USA) with atmospheric pressure chemical ionization. The column and autosampler temperatures were 35°C and 10°C, respectively. The separation was performed on an CAPCELL PAK NH2 UG80 (5 *μ*m, 4.6 mm I.D.×250 mm L., Shiseido Co., Ltd., Tokyo, Japan) using a mixture of 0.1% dichloromethane-acetonitrile/water (90/10, v/v) at a flow rate of 1 mL/min. The analytical run time was 18 min. The monitored ion was used for 217 m/z → 181 m/z for sorbitol and 215 m/z → 179 m/z for fructose.

## 3. Results

### 3.1. Body Weight, Plasma Glucose, and Glycemic Hemoglobin

**Figure 1:**
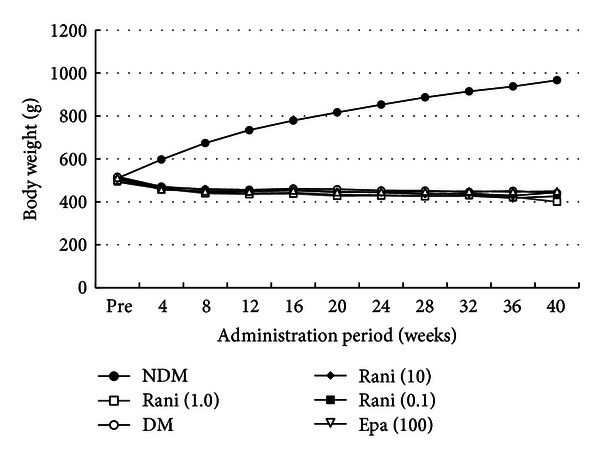
Body weight during the experiment. The SD rats are heavier than the SDT rats with or without treatment. NDM: non-diabetes mellitus; DM: diabetes mellitus; rani: ranirestat; epa: epalrestat.

**Figure 2:**
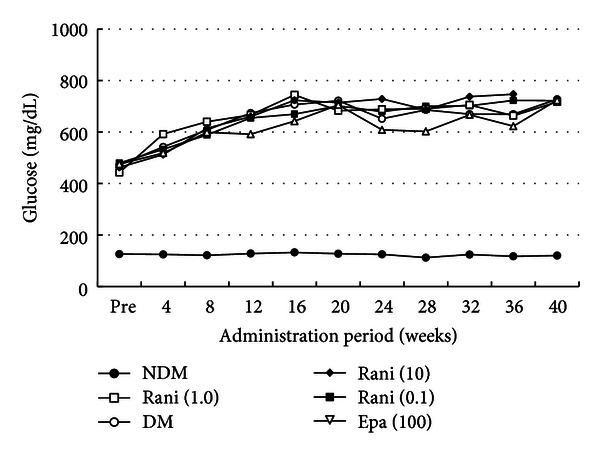
Plasma glucose levels during the experiment. The mean plasma glucose levels of the SD rats are significantly lower than those of the SDT rats with or without treatment. There is no significant difference in the plasma levels among the SDT rats with or without treatment. NDM: non-diabetes mellitus; DM: diabetes mellitus; rani: ranirestat; epa: epalrestat.

**Figure 3:**
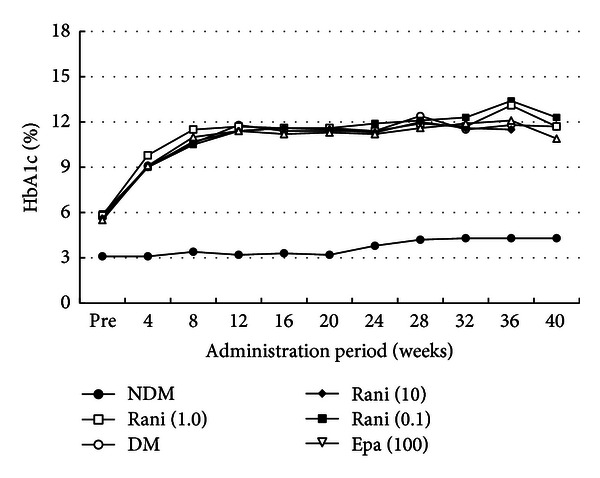
HbA1c levels during the experiment. The mean HbA1c levels of the SD rats are significantly lower than those of the SDT rats with or without treatment. There is no significant difference in the HbA1c levels among the SDT rats with or without treatment. NDM: non-diabetes mellitus; DM: diabetes mellitus; rani: ranirestat; epa: epalrestat.

Figures 1, 2, and 3 show the changes in weight, plasma glucose, and HbA1c during the experiment. Compared with the SD rats, the SDT rats were significantly (*P* < 0.01) lighter with or without ARI treatment. The mean plasma glucose levels and HbA1c levels of the SDT rats were significantly (*P* < 0.01) higher than those of the SD rats. However, there was no significant difference in the blood glucose levels and HbA1c levels among the SDT rats with or without treatment. The ARIs did not affect the glycemic control. Therefore, we did not consider the glycemic effect in this study.

### 3.2. Prevalence of Cataract

**Figure 4:**
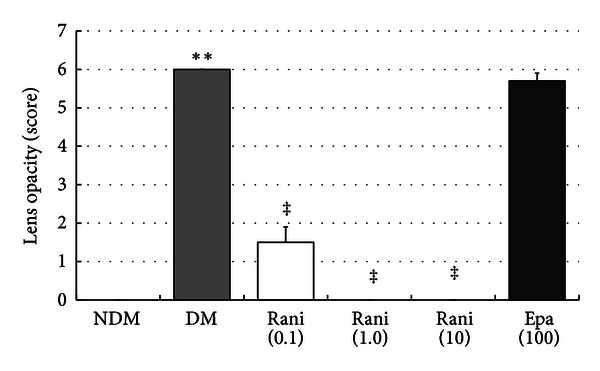
The total cataract scores at 15 weeks. The total cataract score of the untreated SDT rats at 15 weeks is significantly higher than that of the SD rats. **P < 0.01, Wilcoxon rank sum test. The total cataract score of the SDT rats treated with ranirestat at 15 weeks is significantly lower than that of the untreated SDT rats. ^‡^ *P* < 0.01, Wilcoxon rank sum test. There is no significant difference in the total cataract score between the untreated SDT rats and the SDT rats treated with epalrestat. NDM: non-diabetes mellitus; DM: diabetes mellitus; rani: ranirestat; epa: epalrestat.

**Figure 5:**
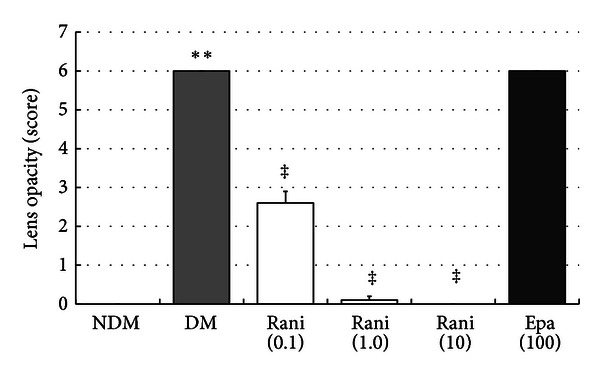
The total cataract scores at 40 weeks. The total cataract score of the untreated SDT rats at 40 weeks is significantly higher than that of the SD rats. **P < 0.01, Wilcoxon rank sum test. The total cataract score of the SDT rats treated with ranirestat at 40 weeks is significantly lower than that of the untreated SDT rats. ^‡^ *P* < 0.01, Wilcoxon rank sum test. There is no significant difference in the total cataract score between the untreated SDT rats and the SDT rats treated with epalrestat. NDM: non-diabetes mellitus; DM: diabetes mellitus; rani: ranirestat; epa: epalrestat.

Figures 4 and 5 show the total cataract scores at 15 and 40 weeks. The incidence of cataracts at 15 weeks was greater in the untreated SDT rats (6.0 ± 0.0) compared with the normal SD rats (0.0 ± 0.0) (*P* < 0.01, Wilcoxon rank-sum test). Fewer cataracts developed in the ranirestat-treated SDT rats at 15 weeks (1.5 ± 0.4 for 0.1 mg/kg/day; 0.0 ± 0.0, for 1 mg/kg/day; 0.0 ± 0.0, for 10 mg/kg/day) than in untreated SDT rats (*P* < 0.01, Wilcoxon rank-sum test). Epalrestat (5.7 ± 0.2 for 100 mg/kg/day) did not prevent cataract development in SDT rats. Similarly, the incidence of cataracts at 40 weeks was greater in the untreated SDT rats (6.0 ± 0.0) compared with the normal SD rats (0.0 ± 0.0) (*P* < 0.01, Wilcoxon rank-sum test). Fewer cataracts developed in the ranirestat-treated SDT rats at 40 weeks (2.6 ± 0.3 for 0.1 mg/kg/day; 0.1 ± 0.1, for 1 mg/kg/day; 0.0 ± 0.0, for 10 mg/kg/day) than in untreated SDT rats (*P* < 0.01, Wilcoxon rank-sum test). Epalrestat (6.0 ± 0.0 for 100 mg/kg/day) did not prevent cataract development in SDT rats.

### 3.3. MNCV in the Sciatic Nerve

**Figure 6:**
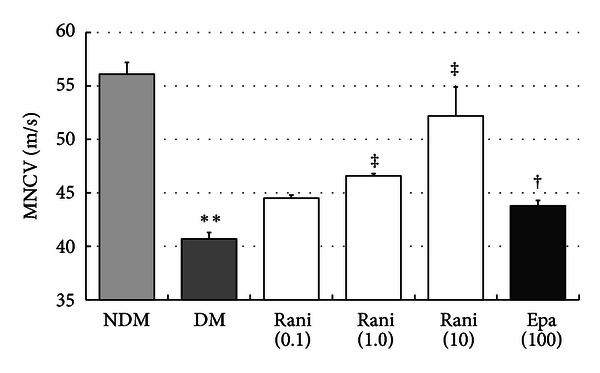
The MNCV levels in the sciatic nerves at 40 weeks. The MNCV levels in the sciatic nerves of the untreated SDT rats at 40 weeks are significantly lower than those of the SD rats. **P < 0.01, Student's *t*-test. The MNCV levels in the sciatic nerves of the SDT rats treated with ranirestat and those treated with epalrestat are significantly higher than those of the untreated SDT rats. ^‡^ *P* < 0.01, Dunnett type mean rank test and ^†^ *P* < 0.05, Student's *t*-test. NDM: non-diabetes mellitus; DM: diabetes mellitus; rani: ranirestat; epa: epalrestat.

Ranirestat (44.5 ± 0.3 m/s for 0.1 mg/kg/day, *P* = 0.274; 46.6 ± 0.2 m/s for 1 mg/kg/day, *P* < 0.01; 52.2 ± 2.7 m/s for 10 mg/kg/day, *P* < 0.01, *n* = 5–7, respectively; Dunnett's test) prevented the decrease in MNCV in the sciatic nerves at 40 weeks observed in the control SDT rats (40.7 ± 0.6 m/s) (*n* = 5) in a dose-dependent manner. Epalrestat (100 mg/kg/day) also prevented a decrease in MNCV (43.8 ± 0.5 m/s, *n* = 4, *P* < 0.05 by *t*-test). The MNCV in the non-diabetic control SD group was 56.1 ± 1.1 m/sec (*n* = 6) (Figure 6). Thus, ranirestat and epalrestat both prevented development of DN in SDT rats.

### 3.4. Sorbitol and Fructose in Lenses

**Figure 7:**
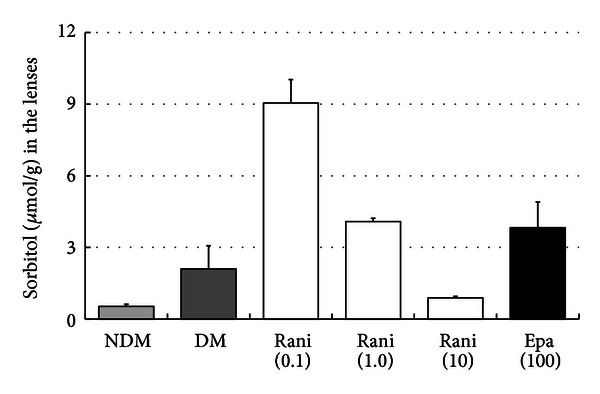
The sorbitol levels in the lenses at 15 weeks. Although there seems to be a dose-dependent decrease in the sorbitol levels in the lenses with administration of ranirestat, the sorbitol levels in the untreated lenses and in the lenses treated with epalrestat are very low. NDM: non-diabetes mellitus; DM: diabetes mellitus; rani: ranirestat; epa: epalrestat.

**Figure 8:**
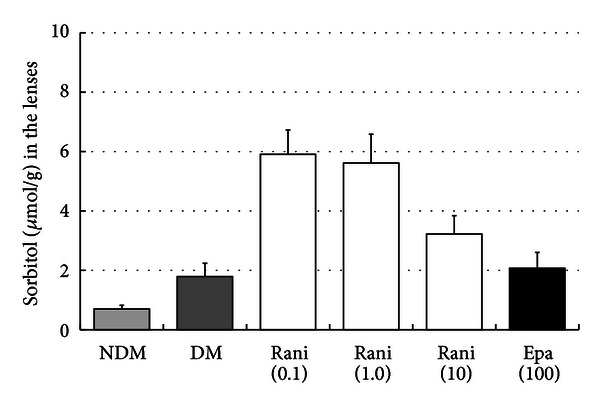
The sorbitol levels in the lenses at 40 weeks. Although there seems to be a dose-dependent decrease in the sorbitol levels in the lenses with administration of ranirestat, the sorbitol levels in the untreated lenses and in those treated with epalrestat are very low. NDM: non-diabetes mellitus; DM: diabetes mellitus; rani: ranirestat; epa: epalrestat.

**Figure 9:**
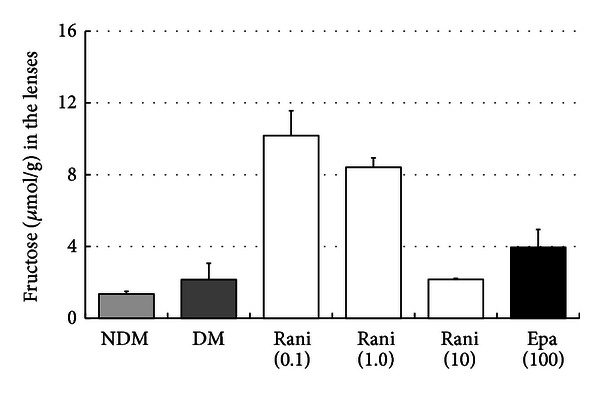
The fructose levels in the lenses at 15 weeks. Although there seems to be a dose-dependent decrease in the fructose levels in the lenses with administration of ranirestat, the fructose level in the untreated lenses is very low. NDM: non-diabetes mellitus; DM: diabetes mellitus; rani: ranirestat; epa: epalrestat.

**Figure 10:**
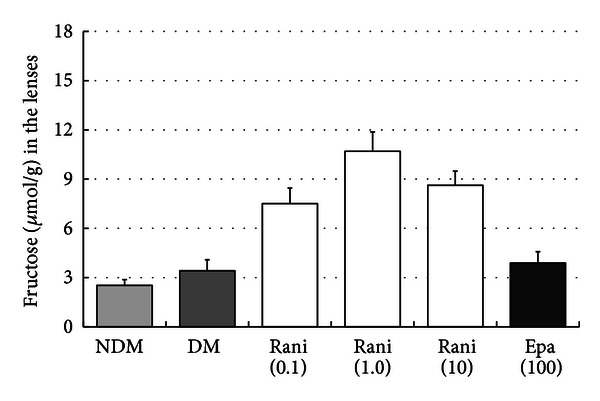
The fructose levels in the lenses at 40 weeks. The fructose level in the untreated lenses is very low compared with other ARI-treated lenses. NDM: non-diabetes mellitus; DM: diabetes mellitus; rani: ranirestat; epa: epalrestat.

The sorbitol and fructose levels in the lenses at 15 and 40 weeks are shown in Figures 7, 8, 9, and 10. Although there seems to be a dose-dependent decrease in sorbitol and fructose in the lenses resulting from treatment with ranirestat, the levels in the untreated lenses were also very low. 

### 3.5. Sorbitol and Fructose in Sciatic Nerves

**Figure 11:**
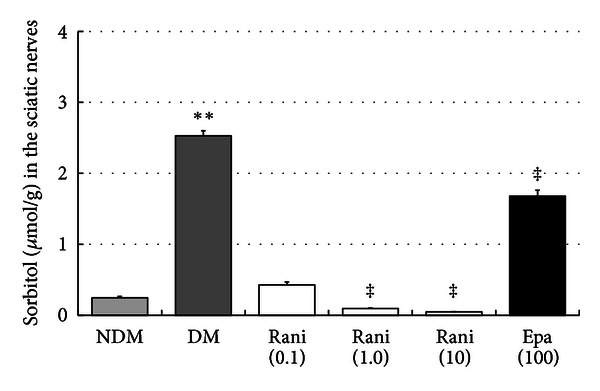
The sorbitol levels in the sciatic nerves at 15 weeks. The sorbitol level in the sciatic nerves in the untreated SDT rats is significantly higher that of the SD rats. **P < 0.01, Student's *t*-test. The sorbitol levels in the sciatic nerves treated with ranirestat (1.0 and 10 mg/kg/day) are significantly lower than those in the untreated SDT rats. ^‡^ *P* < 0.01, Dunnett type joint-ranking test. The sorbitol level in the sciatic nerves treated with epalrestat is also significantly lower than in the untreated SDT rats. ^‡^ *P* < 0.01, Student's *t*-test. NDM: non-diabetes mellitus; DM: diabetes mellitus; rani: ranirestat; epal: epalrestat.

**Figure 12:**
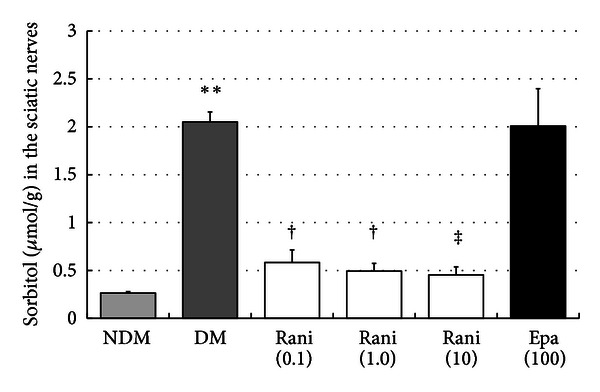
The sorbitol levels in the sciatic nerves at 40 weeks. The sorbitol level in the sciatic nerves in the untreated SDT rats is significantly higher that of the SD rats. **P < 0.01, Student's *t*-test. The sorbitol levels in the sciatic nerves treated with all doses of ranirestat (10.0 and 1.0 mg/kg/day) are lower than in the untreated SDT rats. ^†^ *P* < 0.05 and ^‡^ *P* < 0.01, Dunnett type joint-ranking test. Epalrestat does not affect the reduction in the sorbitol level. NDM: non-diabetes mellitus; DM: diabetes mellitus; rani: ranirestat; epal: epalrestat.

 The sorbitol levels in the sciatic nerves at 15 and 40 weeks are shown in Figures 11 and 12. The increase in the sorbitol level in the sciatic nerves of the control rats (2.05 ± 0.10 nmol/g) decreased significantly (*P* < 0.01, Dunnett's test) with administration of ranirestat (10.0 and 1.0 mg/kg/day) and epalrestat (100 mg/kg/day) at 15 weeks. At 40 weeks, the increase in the sorbitol level in the sciatic nerves in the control rats (2.05 ± 0.10 nmol/g) decreased significantly with all doses of ranirestat (0.1 and 1.0 mg/kg/day, *P* < 0.05; 10.0 mg/kg/day, *P* < 0.01, Dunnett's test). However, epalrestat did not have a significant effect on the increased sorbitol level.

**Figure 13:**
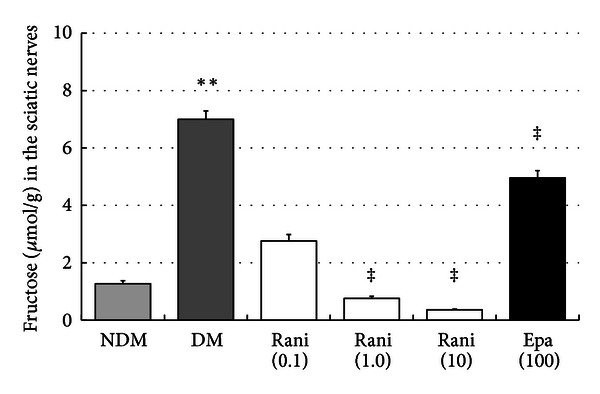
The fructose levels in the sciatic nerves at 15 weeks. The fructose level in the sciatic nerves of the untreated SDT rats is significantly higher that of the SD rats. **P < 0.01, Student's *t*-test. The fructose levels in the sciatic nerves treated with ranirestat (1.0 and 10 mg/kg/day) are significantly lower than in the untreated SDT rats. ^‡^ *P* < 0.01, Dunnett type joint-ranking test. The fructose levels in the sciatic nerves treated with epalrestat are also significantly lower than in the untreated SDT rats. ^‡^ *P* < 0.01, Student's *t*-test. NDM: non-diabetes mellitus; DM: diabetes mellitus; rani: ranirestat; epal: epalrestat.

**Figure 14:**
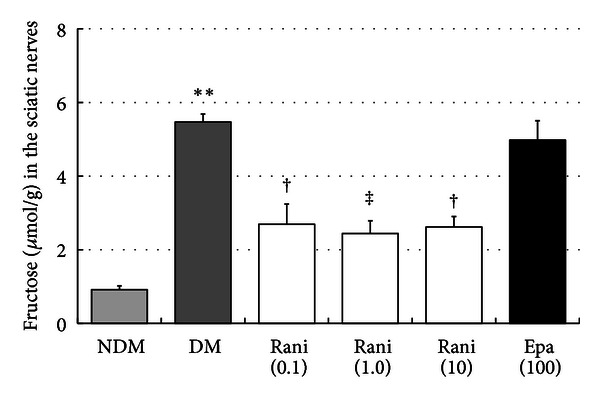
The fructose levels in the sciatic nerves at 40 weeks. The fructose levels in the sciatic nerves in the untreated SDT rats are significantly higher in the SD rats. **P < 0.01, Student's *t*-test. The fructose levels in the sciatic nerves treated with all doses of ranirestat are significantly lower than in the untreated SDT rats. ^†^ *P* < 0.05 and ^‡^ *P* < 0.01, Dunnett type joint-ranking test. Epalrestat does not affect the reduced fructose levels. NDM: non-diabetes mellitus; DM: diabetes mellitus; rani: ranirestat; epal: epalrestat.

The fructose levels in the sciatic nerves at 15 and 40 weeks are shown in Figures 13 and 14. The increase in the fructose levels in the sciatic nerves of the control rats was decreased significantly (*P* < 0.05, Dunnett's test) by ranirestat (1.0 and 10.0 mg/kg/day) and epalrestat (100 mg/kg/day) at 15 weeks. At 40 weeks, the increased level of fructose in the sciatic nerves in the control rats decreased significantly with all doses of ranirestat (0.1 and 10.0 mg/kg/day, *P* < 0.05; 1.0 mg/kg/day, *P* < 0.01, Dunnett's test). However, epalrestat did not have a significant effect on the fructose level.

### 3.6. Histopathology

**Figure 15:**
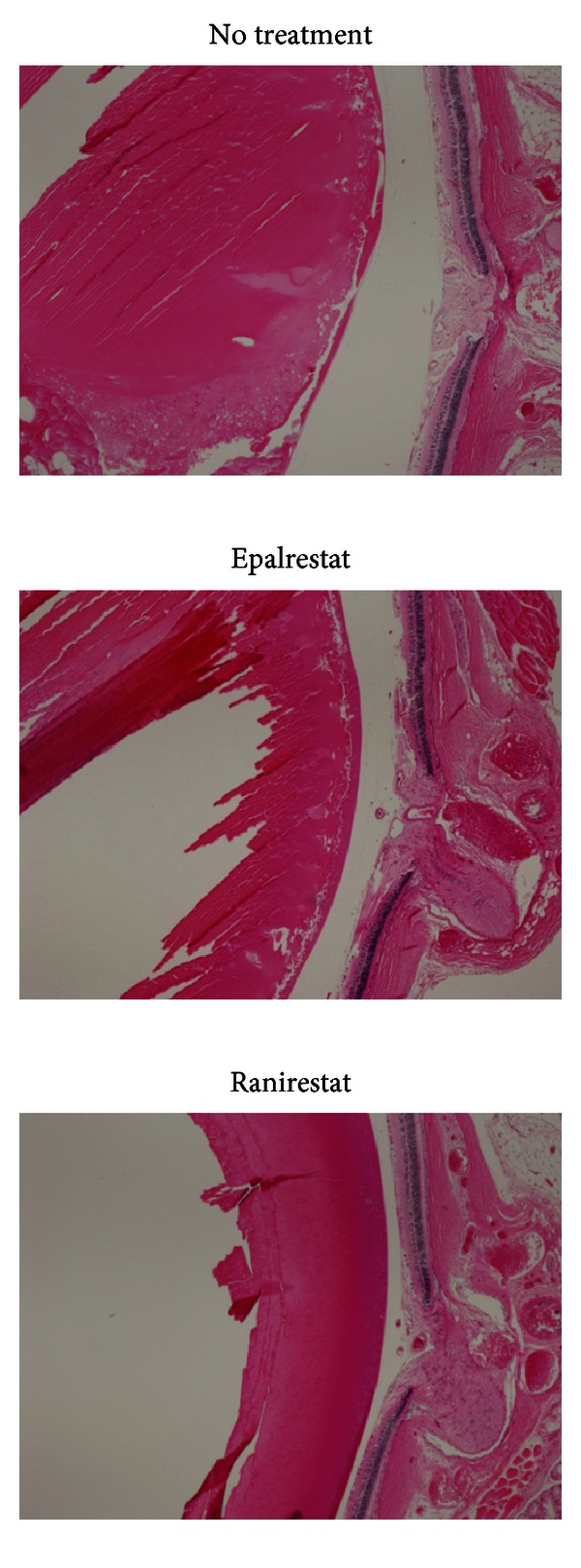
Pathology (hematoxylin, original magnification 4x) at 40 weeks. In an untreated eye (left), the lens has a sclerotic nucleus floating in liquefied cortex and vacuolation and disintegration of cortex fibers. In the epalrestat-treated eye (middle), the findings are virtually the same as in the untreated eye. In the ranirestat-treated eyes (right), the eye has an almost normal lens and retina.

**Figure 16:**
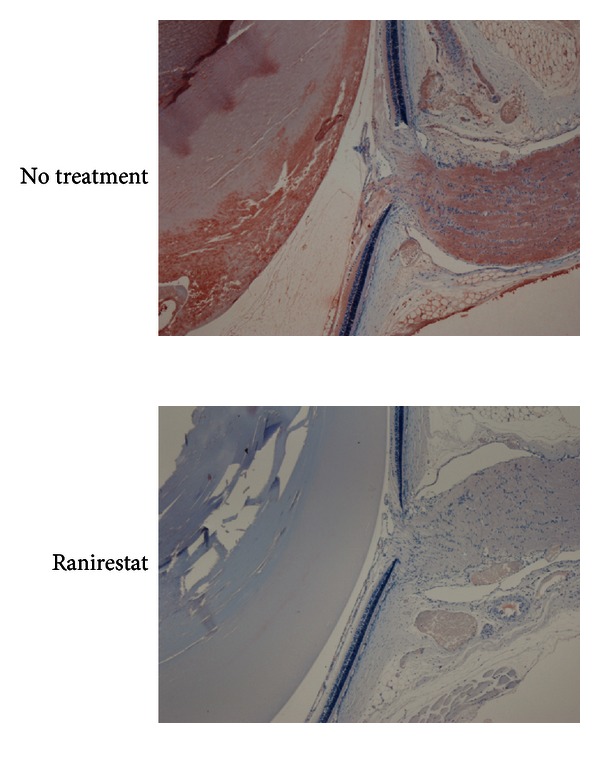
Immunostaining for CML (original magnification 4x) at 40 weeks. Immunostaining for CML shows severe staining in an untreated eye (top) compared with minimal staining in a ranirestat-treated eye (bottom).

Standard hematoxylin staining of untreated eyes (Figure 15, left) showed a sclerotic nucleus floating in liquefied cortex and vacuolation and disintegration of cortex fibers. Epalrestat-treated eyes (Figure 15, middle) had almost the same findings as untreated eyes. However, ranirestat-treated eyes (Figure 15, right) had almost normal lens findings. Immunostaining for CML showed severe staining in the untreated eyes (Figure 16, top) compared with minimal staining in the ranirestat-treated (10 mg/kg/day for 40 weeks) lenses (Figure 16, bottom). The lens cortex in the untreated eyes showed liquefaction and extensive staining for CML. However, the treated eyes had minimal staining for CML in the lens nucleus and cortex.

## 4. Discussion

We previously reported a preventive effect of ranirestat on DN and cataract in streptozotocin (STZ)-induced diabetic rat [18]. In that study, ranirestat reduced sorbitol accumulation in the sciatic nerve and improved the decrease in MNCV in STZ-induced diabetic rats. The drug also had a potent preventative effect on lens opacity throughout the experimental period. 

However, the STZ-induced diabetic rat is not an ideal animal model of diabetic ocular complications because it usually shows only the early phase of DR and no rubeosis. The STZ-induced diabetic rat shows cataract but the SDT rat develops a more advanced hypermature cataract and three major diabetic ocular complications, proliferative DR, cataract, and rubeosis [19]. The cataract develops to hypermaturity, and DR develops to the advanced stage. Large retinal folds mimicking a tractional retinal detachment with extensive fluorescein leakage around the optic disc are the most characteristic findings of DR in this rat. In some SDT rats, rubeosis develops with a massive hemorrhage in the anterior chamber associated with neovascular fibrous membranes around the iris. Although there are some differences in the diabetic ocular complications between SDT rats and human patients with diabetes, the ocular complications in SDT rats mimic those in humans. Thus, the SDT rat is useful for conducting research into diabetic ocular complications. Using this unique rat model, we tested the effect of ranirestat, a new ARI, on DR in SDT rats in a previous study. Ranirestat prevented development of proliferative DR in SDT rats [17]. We then conducted the current experiment to evaluate the effect of ranirestat on cataracts and DN in SDT rats and to compare the effect of the commercially available ARI epalrestat. The clinical dose of eparlestat is 150 mg/day/adult. According to this dose, about 2 mg/kg/day may be appropriate to obtain the ARI effect. Therefore, we tested three different doses of ranirestat (0.1, 1, and 10 mg/kg/day) and a hyperdose of epalrestat (100 mg/kg/day). The untreated SDT rats and the epalrestat-treated SDT rats developed mature cataracts. However, minimal cataracts developed in the ranirestat-treated SDT rats. We think that this difference results from the differences in tissue penetration of each ARI. Therefore, high sorbitol and fructose levels may be expected in the lenses of the untreated SDT rats and epalrestat-treated SDT rats as in sciatic nerves but low levels in ranirestat-treated SDT rats. However, the results were not what we expected. We previously reported similar findings that the sorbitol level in the lenses of untreated diabetic rats decreases with time [20]. Advanced disruption and disintegration of the lens cortex were observed in advanced mature cataracts of the untreated SDT rats (Figure 15). This pathological change might cause sorbitol and fructose to leak out of the lens capsule possibly because of a minor capsular rupture with time. The pathological change also might have induced another metabolic change in the advanced stage of the polyol pathway. Fructose decomposes to 3-deoxyglucosone and sorbitol and fructose eventually run out of the closed lens capsule. If the levels of sorbitol and fructose in the lens were measured in the early phase of diabetic cataract, the results might have been different.

Ranirestat prevented the decrease in MNCV in the sciatic nerve in a dose-dependent manner, and the effect was stronger than that with epalrestat (Figure 6). 

In the sciatic nerve, significant decreases in sorbitol and fructose resulting from treatment with ranirestat were seen (Figures 11, 12, 13, and 14) compared with high levels of sorbitol and fructose in untreated diabetic SDT rats, which must be associated with the decrease in MNCV. 

We believe that our findings strongly suggest a potential therapeutic use for ranirestat in the prevention of diabetic ocular and neural complications. Epalrestat, the only commercially available ARI in the world, also was confirmed to be effective for DN, but its effect was somehow limited for diabetic ocular complications. Thus, it appears that ARIs have a preventive effect on diabetic complications, and ranirestat may prevent diabetic ocular complications more effectively compared with epalrestat, which had limited beneficial effects.
